# Common Cholinergic, Noradrenergic, and Serotonergic Drugs Do Not Block VNS-Mediated Plasticity

**DOI:** 10.3389/fnins.2022.849291

**Published:** 2022-02-23

**Authors:** Robert A. Morrison, Stephanie T. Abe, Tanya Danaphongse, Vikram Ezhil, Armaan Somaney, Katherine S. Adcock, Robert L. Rennaker, Michael P. Kilgard, Seth A. Hays

**Affiliations:** ^1^School of Behavioral and Brain Sciences, University of Texas at Dallas, Richardson, TX, United States; ^2^Texas Biomedical Device Center, University of Texas at Dallas, Richardson, TX, United States; ^3^Erik Jonsson School of Engineering and Computer Science, University of Texas at Dallas, Richardson, TX, United States

**Keywords:** vagus nerve stimulation (VNS), neuroplasticity, rehabilitation, motor cortex, neuromodulation, acetylcholine, norepinephrine, serotonin

## Abstract

Vagus nerve stimulation (VNS) delivered during motor rehabilitation enhances recovery from a wide array of neurological injuries and was recently approved by the U.S. FDA for chronic stroke. The benefits of VNS result from precisely timed engagement of neuromodulatory networks during rehabilitative training, which promotes synaptic plasticity in networks activated by rehabilitation. Previous studies demonstrate that lesions that deplete these neuromodulatory networks block VNS-mediated plasticity and accompanying enhancement of recovery. There is a great deal of interest in determining whether commonly prescribed pharmacological interventions that influence these neuromodulatory networks would similarly impair VNS effects. Here, we sought to directly test the effects of three common pharmaceuticals at clinically relevant doses that target neuromodulatory pathways on VNS-mediated plasticity in rats. To do so, rats were trained on a behavioral task in which jaw movement during chewing was paired with VNS and received daily injections of either oxybutynin, a cholinergic antagonist, prazosin, an adrenergic antagonist, duloxetine, a serotonin-norepinephrine reuptake inhibitor, or saline. After the final behavioral session, intracortical microstimulation (ICMS) was used to evaluate reorganization of motor cortex representations, with area of cortex eliciting jaw movement as the primary outcome. In animals that received control saline injections, VNS paired with training significantly increased the movement representation of the jaw compared to naïve animals, consistent with previous studies. Similarly, none of the drugs tested blocked this VNS-dependent reorganization of motor cortex. The present results provide direct evidence that these common pharmaceuticals, when used at clinically relevant doses, are unlikely to adversely impact the efficacy of VNS therapy.

## Introduction

Vagus nerve stimulation (VNS) paired with rehabilitation has emerged as a therapeutic strategy to enhance recovery in a range of neurological disorders, including stroke (Khodaparast et al., 2013, 2014; Hays et al., 2014b,^2016^; Dawson et al., 2016, 2021; Kilgard et al., 2018; Kimberley et al., 2018; Meyers et al., 2018; Pruitt et al., 2021), traumatic brain injury (Pruitt et al., 2016b), neuropathy (Meyers et al., 2019; Darrow et al., 2020a,^2021^), spinal cord injury (SCI; Ganzer et al., 2018; Darrow et al., 2020b; Kilgard et al., 2021), and post-traumatic stress disorder (PTSD; George et al., 2008; Pena et al., 2014; Noble et al., 2017; Kilgard et al., 2020; Souza et al., 2020). Following a recently completed pivotal study, VNS paired with rehabilitation has received United States FDA approval as the first neuromodulation therapy for chronic stroke (Dawson et al., 2021; FDA, 2021).

VNS-dependent enhancement of recovery is attributed to synaptic plasticity in central networks activated by rehabilitation (Porter et al., 2012; Hulsey et al., 2016, 2019; Meyers et al., 2018; Morrison et al., 2019, 2021; Tseng et al., 2020). VNS rapidly activates cholinergic, noradrenergic, and serotonergic systems (Dorr, 2006; Roosevelt et al., 2006; Nichols et al., 2011; Hulsey et al., 2017). Coincident release of these pro-plasticity neuromodulators coupled with neural activity during rehabilitation promotes synaptic plasticity in task-specific activated circuits, leading to the strengthening of pathways mediating recovery (Dorr, 2006; Roosevelt et al., 2006; Seol et al., 2007; He et al., 2015; Hulsey et al., 2017).

As VNS-based therapies begin to translate into clinical studies and clinical practice, there is a growing interest in evaluating factors that may influence therapeutic efficacy. Individuals that may benefit from VNS-paired rehabilitation are commonly prescribed medications for issues directly stemming from their specific neurological injuries, related comorbidities, or other unrelated conditions. For example, one of the most common disabilities after SCI is urinary incontinence, for which the anti-cholinergic muscarinic receptor antagonist oxybutynin is often prescribed. Prazosin, an adrenergic alpha-receptor antagonist, is commonly prescribed for hypertension after stroke and to alleviate sleep disturbances in those with PTSD. Duloxetine, a serotonin norepinephrine reuptake inhibitor (SNRI) used to treat major depressive disorder, generalized anxiety disorder, and neuropathic pain, is commonly prescribed to treat symptoms of stroke, neuropathy, SCI, and PTSD.

All of these medications share a common feature: they target neuromodulatory systems driven by VNS therapy. Function of the cholinergic system is critical for VNS-mediated plasticity, as total depletion of acetylcholine in the forebrain prevents VNS-mediated plasticity (Hulsey et al., 2016) and consequently enhancement of recovery (Meyers et al., 2019). Similarly, depletion of noradrenergic and serotonergic transmission in central networks prevents VNS-dependent enhancement of plasticity (Hulsey et al., 2019). Based on their mechanisms of action, it is possible that medications that target these neuromodulatory networks may blunt the efficacy of VNS therapy in patients. Alternatively, the common clinical doses of these medications may not reach a level high enough to substantially impair VNS-dependent effects on the central nervous system. Because these medications are often crucial to maintaining quality of life for those who take them, a clear understanding of how clinically relevant doses of these drugs is critical to the clinical implementation of VNS-based therapies. Here, we sought to directly address this point by testing the effects of several common drugs at clinically relevant doses on VNS-mediated plasticity.

## Materials and Methods

### Subjects

Fifty-one female Sprague Dawley rats weighing approximately 250 grams were used in this study (Charles River Labs, Wilmington, MA, United States). All rats were housed in a reversed 12:12 h light-dark cycle. Rats that underwent behavioral training were food restricted on weekdays during shaping and training with *ad libitum* access to food on weekends. All rats were maintained at or above 85% body weight. All handling, housing, stimulation, and surgical procedures were approved by The University of Texas at Dallas Institutional Animal Care and Use Committee.

### Behavioral Training

**FIGURE 1:**
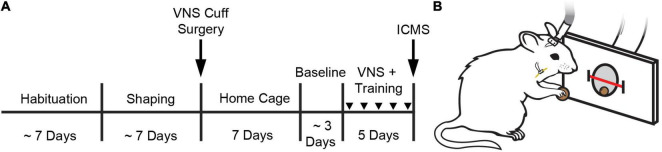
Behavioral task and experimental design. **(A)** Experimental timeline. **(B)** Illustration of a rat performing the behavioral task. A stimulating cable plugged into a headmount-connector, the subcutaneous stimulation leads and nerve cuff, and the vagus nerve are shown. A feeder dispenses food pellets into a nosepoke and an infrared beam monitors movement into and out of the nosepoke. Stimulation occurs no faster than once every 8 s. Groups received VNS paired with behavioral training and daily injections of either oxybutynin (Oxy), prazosin (Praz), duloxetine (Dulox), or saline (Veh). Injections were given 1 h before the start of daily training. Injections during training are indicated by downward facing triangles.

Rats were trained on a simple automated behavioral task that allowed triggering of VNS during chewing (Morrison et al., 2020, 2021; Figure 1A). The behavioral training apparatus consisted of an acrylic cage with a nosepoke food dispenser at one end. A food pellet (45 mg dustless precision pellet, BioServ, Frenchtown, NJ, United States) was delivered to the food dispenser (Figure 1B). An infrared beam sensor positioned in the food dispenser was used to determine when the rat entered the nosepoke to retrieve the food pellet. Upon breaking the infrared beam, another pellet was dispensed after an 8 s delay. Additionally, in the appropriate groups, VNS was triggered 3 s after beam break. This stimulation timing results in reliable delivery of VNS during chewing (Morrison et al., 2020). Each behavioral session continued until either 100 pellets had been dispensed, or until 1 h had elapsed. Rats received a supplement of approximately 100 food pellets if they did not receive at least 100 pellets in a day to maintain weight.

Rats performed the task twice per day, 5 days per week, with daily sessions separated by at least 1 h. Rats were trained on the task until they reliably consumed 100 pellets within 1 h each session. Rats were then implanted with a VNS cuff and recovered for 7 days in their home cage with *ad libitum* access to food and water. Seven days after surgery, rats were randomly allocated to one of four groups to receive 10 additional training sessions over 5 days. During these additional training sessions, groups received VNS and daily injections of either oxybutynin, prazosin, duloxetine, or saline. Drug or vehicle injections were delivered approximately 1 h before first behavioral training session. Twenty-four hours after the conclusion of behavioral training, all rats underwent ICMS motor cortex mapping (Figure 1A).

### Surgical Implantation

All surgeries were performed using aseptic technique under general anesthesia. Rats were implanted with a stimulating cuff on the left cervical vagus nerve as described in previous studies (Khodaparast et al., 2013, 2014; Hays et al., 2014a,b; Morrison et al., 2019). Rats were anesthetized with ketamine hydrochloride (50 mg/kg, i.p.), xylazine (20 mg/kg, i.p.), and acepromazine (5 mg/kg, i.p.), and were placed in a stereotaxic apparatus. An incision was made down the midline of the head to expose the skull. Bone screws were inserted into the skull at points surrounding the lamboid suture and over the cerebellum. A two-channel connector was mounted to the screws using acrylic. The rat was then removed from the stereotactic apparatus and placed in a supine position.

An incision was made on the left side of the neck and the overlying musculature was blunt dissected to reveal the left cervical vagus nerve. The nerve was gently dissected away from the carotid artery. A cuff electrode was implanted surrounding the vagus nerve, and the cuff was closed with a suture knot around the nerve, securing it in place. Leads were tunneled subcutaneously to connect with the two-channel connector mounted on the skull. Nerve activation was confirmed by observation of a ≥5% drop in blood oxygen saturation in response to a 10 s stimulation train of VNS, as in previous studies (Morrison et al., 2019, 2020, 2021). The head and neck incisions were then sutured, and rats received subcutaneous injections of 4 mL 50:50 0.9% saline 5% dextrose solution. A 7 day recovery period followed surgery during which animals did not perform behavioral training. All behaviorally trained rats underwent implantation procedures. Consistent with previous studies, the bipolar, circumferential nerve cuff was composed of a 2.5 mm long, 1 mm inner diameter polyurethane tube. Two platinum-iridium contact electrodes with 270 degrees of coverage spaced approximated 1 mm apart were affixed inside the nerve cuff (Rios et al., 2019; Sanchez et al., 2020).

### Vagus Nerve Stimulation

Upon return to behavioral testing after surgery, rats were randomly assigned to groups to receive motor training paired with VNS and daily injections of oxybutynin (*n* = 8), prazosin (*n* = 8), duloxetine (*n* = 8), or saline (*n* = 9). In the initial sessions after implantation, no stimulation was delivered in any group while rats were allowed to acclimate to being attached to stimulating cables until they reliably consumed 100 pellets in a 1 h session. Acclimation lasted approximately 3 ± 2 days. Once acclimated, rats then underwent 5 days of training and received treatment according to their group. VNS was triggered 3 s after nosepoke beam break once a pellet had been dispensed during behavioral training, resulting in stimulation that was consistently delivered during chewing of the pellet (Morrison et al., 2020). Each 0.5 s stimulation train consisted of 100 μs biphasic pulses delivered at 30 Hz at an intensity 0.8 mA, parameters known to maximize VNS-mediated effects (Borland et al., 2018; Loerwald et al., 2018; Morrison et al., 2020; Pruitt et al., 2021). A digital oscilloscope (PicoScope 2204A, PP906, Pico Technology, St Neots, United Kingdom) was used to monitor voltage across the electrodes during each stimulation to ensure cuff functionality.

### Drug Administration and Dosage

We surveyed several rodent studies using oxybutynin (Oka et al., 2001; Angelico et al., 2005; Aizawa et al., 2012; Wada et al., 2017), prazosin (Lê et al., 2011; Verplaetse et al., 2012; Do Monte et al., 2013), and duloxetine (Molteni et al., 2009; Miyazaki and Yamamoto, 2012; Di Cesare Mannelli et al., 2017; Pérez et al., 2018; Yoneda et al., 2020) to determine appropriate dosage. Doses were selected based on reported ranges that produced intended pharmacological effects but were below levels found to cause analogous unwanted effects observed in clinical use. All drugs have plasma half-lives that fall within the timing of behavioral training (Jaillon, 1980; Chae et al., 2013; Tian et al., 2019). Groups received motor training paired with VNS and daily injections of oxybutynin chloride (10 mg/kg, s.c., Fisher Scientific, Hampton, NH, United States – 18604931), prazosin hydrochloride (5 mg/kg, s.c., Fisher Scientific, Hampton, NH, United States – AAJ61712MD), duloxetine (20 mg/kg, s.c., Fisher Scientific, Hampton, NH, United States – D42231G), or saline (s.c.). All drugs were reconstituted in saline and delivered subcutaneously under the skin fold of the back approximately 1 h prior to first behavioral training session each day for 5 days.

### Intracortical Microstimulation Mapping

Approximately 24 h after their last behavioral session, rats underwent ICMS to derive cortical movement representation maps according to standard procedures (Neafsey and Sievert, 1982; Neafsey et al., 1986; Kleim et al., 2003; Porter et al., 2012; Pruitt et al., 2016a; Morrison et al., 2019). Rats were anesthetized with intraperitoneal injections of ketamine hydrochloride (80 mg/kg) and xylazine (10 mg/kg). Rats received supplemental doses of ketamine as necessary throughout the procedure in order to maintain a consistent level of anesthesia as indicated by breathing rate, vibrissae whisking, and toe pinch reflex. Rats were placed in a stereotactic apparatus and a craniotomy and durotomy were performed to expose the left motor cortex (4 mm to −3 mm AP and 0.25 mm to 5 mm ML). To prevent cortical swelling, a small incision was made in the cisterna magna.

A tungsten electrode with an impedance of approximately 0.7 MΩ (UEWMEGSEBN3M, FHC, Bowdoin, ME) was lowered into the brain to a depth of 1.8 mm. Stimulation sites were chosen at random on a grid with sites set 500 μm apart from each other. The next stimulation site was placed at least 1 mm away from the previous site whenever possible. Stimulation consisted of a 40 ms pulse train of 10 monophasic 200 μs cathodal pulses. Stimulation was increased from 10 μA until a movement was observed or until a maximum of 250 μA was reached. The researchers performing ICMS were blinded to experimental group. Movements elicited by stimulation were classified into the following categories: jaw, neck, vibrissa, forelimb, and hindlimb. Prior to ICMS, VNS cuff functionality was confirmed by a stimulation-evoked decrease in blood oxygen saturation in response to a 10 s VNS train, as previously described (Morrison et al., 2019; Rios et al., 2019). All maps from ICMS are included in the supplemental materials (Supplementary Figure 1).

### Subject Exclusion

Forty-one subjects were analyzed in the final results of the study out of a total of 51 subjects. Of the 10 subjects excluded from final analysis, 1 subject was removed due to a non-functional stimulating cuff (indicated by digital oscilloscope readings exceeding 40 V peak-to-peak), 1 subject was excluded due to mechanical headcap failure, 7 subjects died during VNS surgery, and 1 subject died during ICMS surgery. All exclusions were made before collection of ICMS data and are thus unlikely to introduce bias. Additionally, exclusion criteria were predefined before beginning data collection and are consistent with previous studies (Porter et al., 2012; Hulsey et al., 2016, 2019; Morrison et al., 2019, 2020, 2021).

### Statistics

The primary outcome of this study was total area of motor cortex eliciting jaw movements as a result of ICMS. All other movement representations were analyzed as secondary outcome measures. One-way and two-way ANOVA were used to identify differences across groups, as appropriate. *Post hoc* unpaired two-tailed *t*-tests using a Bonferroni-corrected alpha of 0.0125 were used to determine statistically significant differences between individual groups, as appropriate. Statistical tests for each comparison are noted in the text. All data are reported as mean ± SEM.

## Results

### Vagus Nerve Stimulation-Paired Motor Training Enhances Plasticity

**FIGURE 2:**
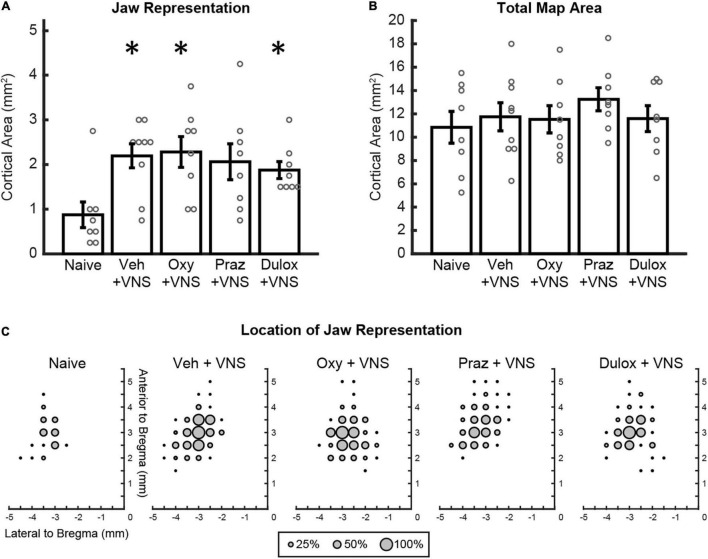
Clinically relevant doses of oxybutynin, prazosin, and duloxetine do not interfere with VNS-mediated plasticity. **(A)** VNS and daily injections of oxybutynin, duloxetine, and saline significantly enhanced jaw representation compared to naïve subjects. Injections of oxybutynin, prazosin, and duloxetine did not significantly reduce jaw representation compared to saline in animals that received VNS paired with training. **(B)** No change in total motor cortex area was observed between groups. **(C)** Average plots of the location of jaw movements for all animals during ICMS. Bars represent mean ± SEM. “*” indicates *p* < 0.0125 compared to naïve.

We first sought to confirm previous observations that pairing VNS with motor training in the absence of drug administration could enhance motor cortex plasticity, but using a vehicle injection to control for the added daily injection procedure. Analysis of electrically stimulated motor cortex area eliciting jaw movement revealed a significant group effect of VNS on motor cortex jaw representation [Two-way ANOVA, *F*(1,39) = 9.91, *p* = 0.003] (Figure 2A). VNS paired with training and injections of saline significantly increased jaw representation compared to naïve animals that did not undergo VNS paired training (Naïve: 0.86 ± 0.29 mm^2^; VNS + Veh: 2.19 ± 0.27 mm^2^, Unpaired *t*-test, *p* = 0.004). These findings replicate previous studies showing that VNS paired with training enhances motor cortex plasticity (Porter et al., 2012; Hulsey et al., 2016, 2019; Morrison et al., 2019, 2020).

### Clinically Relevant Doses of Oxybutynin, Prazosin, and Duloxetine Do Not Interfere With Vagus Nerve Stimulation-Mediated Plasticity

We next sought to investigate whether clinically relevant doses of drugs that target neuromodulatory transmission interfere with VNS-mediated plasticity. In rats that received VNS paired with behavioral training, daily injections of oxybutynin and duloxetine significantly increased jaw representation compared to naïve subjects (Unpaired *t*-test, Naïve: 0.86 ± 0.29 mm^2^, VNS + Oxy: 2.28 ± 0.35 mm^2^, *p* = 0.844; VNS + Dul: 1.88 ± 0.19 mm^2^, *p* = 0.011) (Figure 2A). VNS-paired training and daily injections of prazosin trended toward enhancement of jaw representation compared to naïve subjects but failed to reach significance after correction for multiple comparisons (Unpaired *t*-test, Naïve: 0.86 ± 0.29 mm^2^, VNS + Praz: 2.06 ± 0.40 mm^2^, *p* = 0.031) (Figure 2A). Additionally, administration of oxybutynin, prazosin, and duloxetine did not significantly reduce jaw representation compared to saline in animals that received VNS paired with rehabilitation [Two-way ANOVA, *F*(1,39) = 0.13, *p* = 0.721]. These results suggest that clinically relevant doses of oxybutynin, prazosin, and duloxetine do not directly interfere with VNS-mediated plasticity.

### Behavior, Untrained Movement Representations, and Cortical Excitability Were Not Affected by Drug Administration or Vagus Nerve Stimulation

We next evaluated whether VNS and drug treatment would influence cortical representation of unpaired movements or cortical excitability. Group effects of VNS and drug administration on unpaired movement representations were largely unaffected [Two-way ANOVA: VNS, forelimb: *F*(1,39) = 0.53, *p* = 0.471; vibrissa: *F*(1,39) = 0.42, *p* = 0.519; neck: *F*(1,39) = 7.71, *p* = 0.009; hindlimb: *F*(1,39) = 0.03, *p* = 0.85]. Follow up *post hoc* analysis revealed no significant differences in neck representation between groups (Unpaired *t*-test, Naïve: 0.69 ± 0.23 mm^2^, VNS + Veh: 0.22 ± 0.10, *p* = 0.067; VNS + Oxy: 0.063 ± 0.04 mm^2^, *p* = 0.016; VNS + Praz: 0.28 ± 0.10 mm^2^, *p* = 0.121; VNS + Dul: 0.063 ± 0.04 mm^2^, *p* = 0.016). These results suggest that VNS-mediated synaptic plasticity is specific to the paired movement, consistent with previous studies (Porter et al., 2012; Hulsey et al., 2016, 2019; Morrison et al., 2019, 2020). No significant group effects of drug administration were observed on remaining cortical representations [Two-way ANOVA: Drug, forelimb: *F*(1,39) = 0.046, *p* = 0.971; vibrissa: *F*(1,39) = 0.10, *p* = 0.756; neck: *F*(1,39) = 0.41, *p* = 0.524; hindlimb: *F*(1,39) = 2.44, *p* = 0.127]. VNS with daily injections of oxybutynin, prazosin, and duloxetine did not significantly change average stimulation thresholds required to elicit movement or total motor cortex area during ICMS, indicating no overall effect on cortical excitability [Figure 2B and Supplementary Figure 2A; Stimulation Threshold: One-way ANOVA, *F*(4,36) = 1.24, *p* = 0.311; Total Motor Cortex Area: One-way ANOVA, *F*(4,36) = 0.55, *p* = 0.698]. Group analysis of jaw-specific movement thresholds revealed no significant differences between groups [Supplementary Figure 2B; One-way ANOVA, *F*(4, 36) = 1.22, *p* = 0.321]. Group analysis of lowest-threshold jaw movement coordinates revealed no significant differences between groups [One-way ANOVA, *F*(3,29) = 1.24, *p* = 0.313]. Group analysis of the timing between behavioral training trials revealed no differences in behavior between groups [One-way ANOVA, *F*(3,29) = 0.92, *p* = 0.445]. Furthermore, group analysis of the number of total stimulations given was not significantly different between groups [One-way ANOVA, *F*(3,29) = 1.66, *p* = 0.199]. These results suggest that VNS or treatment with oxybutynin, prazosin, or duloxetine did not alter behavioral performance, motivation, or eating behavior.

## Discussion

Vagus nerve stimulation paired with rehabilitation enhances recovery from a wide array of neurological disorders (Khodaparast et al., 2013, 2014; Hays et al., 2014b,^2016^; Dawson et al., 2016, 2021; Pruitt et al., 2016b,^2021^; Ganzer et al., 2018; Kilgard et al., 2018, 2021; Kimberley et al., 2018; Meyers et al., 2018, 2019; Darrow et al., 2020a,b, 2021) by increasing synaptic plasticity in central networks activated by rehabilitation (Porter et al., 2012; Hulsey et al., 2016, 2019; Meyers et al., 2018; Morrison et al., 2019, 2021; Tseng et al., 2020). This enhancement of synaptic plasticity is mediated by coordinated action of cholinergic, noradrenergic, and serotonergic systems (Dorr, 2006; Roosevelt et al., 2006; Nichols et al., 2011; Hulsey et al., 2017). However, these neuromodulatory systems are commonly targeted by drugs used to treat either direct symptoms of neurological disorders aided by VNS therapy, or common comorbidities in patient populations most likely to receive VNS therapy, raising the possibility that they could negatively impact treatment efficacy. Here, we tested the effects of clinically relevant doses of the cholinergic antagonist, oxybutynin, the adrenergic antagonist, prazosin, and the SNRI, duloxetine, on VNS-mediated plasticity, cortical representation of unpaired movements, and general cortical excitability.

Several studies have demonstrated that blocking neuromodulatory action of cholinergic, serotonergic, and noradrenergic systems can abolish the neural (Hulsey et al., 2019; Meyers et al., 2019) and functional (Meyers et al., 2019) effects of VNS therapy. These studies used immunotoxins that produce virtually complete, long-lasting depletion of neuromodulators. Alternatively, pharmacological manipulations, particularly at commonly utilized levels, typically provide partial and transient actions on neuromodulatory networks. Therefore, we sought to design an experiment using rodent equivalents of doses commonly used in the clinic. To do this, we surveyed several rodent studies using oxybutynin, prazosin, and duloxetine, and selected doses just below those found to cause effects analogous to unwanted effects seen in the clinic, but high enough to cause their intended pharmacological effects.

Oxybutynin acts *via* peripheral action to treat urinary incontinence in the clinic through inactivation of muscarinic receptors. However, at higher doses oxybutynin is known to cross the blood brain barrier (Todorova et al., 2001; Callegari et al., 2011), causing unwanted cognitive effects such as headache, somnolence, dizziness, confusion, and memory impairment (Katz et al., 1998; Chancellor et al., 2012). Therefore, we selected a dose of 10 mg/kg based on previous rodent literature that was known to maximize effects on urinary behavior, while minimizing effects on cognition (Oka et al., 2001; Angelico et al., 2005; Aizawa et al., 2012; Wada et al., 2017).

Prazosin is widely used to treat hypertension after stroke *via* peripheral action on adrenergic receptors, however, in contrast to oxybutynin, it is also prescribed for its effects on central noradrenergic systems as well, often being prescribed for treatment of sleep disturbances and anxiety in those with PTSD. To model this use for prazosin in the rat, we selected a dose of 5 mg/kg, as this dose and lower are known to have effects on sleep continuity, generalized anxiety, and reinstatement behaviors in rat models of PTSD (Lê et al., 2011; Verplaetse et al., 2012; Do Monte et al., 2013).

Duloxetine, used to treat major depressive disorder, generalized anxiety disorder, and neuropathic pain, is also used in the clinic primarily for its central action on noradrenergic and serotonergic systems. To model this use in the clinic, we selected a dose of 20 mg/kg, as this concentration reliably treats symptoms of rodent neuropathic pain in the periphery (Miyazaki and Yamamoto, 2012; Di Cesare Mannelli et al., 2017; Yoneda et al., 2020), and exceeds doses that effect central activity related to models of anxiety and depression (Molteni et al., 2009; Pérez et al., 2018).

With the possibility of VNS therapy reaching an ever-broader patient population, an understanding of how common medications may interact with therapy is of the utmost importance. Identifying drugs that may interfere with VNS action is necessary to avoid blocking the beneficial effects of VNS therapy while allowing patients to maintain dosing of drugs that do not impact VNS. Here, we show that although oxybutynin, prazosin, and duloxetine all act on neuromodulatory systems known to be required for VNS-mediated plasticity and enhancement of recovery, rodent doses that are congruent to those seen prescribed in the clinic do not have significant impact on VNS-mediated effects (Figure 2C). This study suggests that patients undergoing VNS therapy may be able to remain on these drugs without adversely effecting treatment efficacy and is a step toward ensuring maximal therapeutic effect for patients that may benefit from VNS therapy for neurological disorders.

## Data Availability Statement

The original contributions presented in the study are included in the article/Supplementary Material, further inquiries can be directed to the corresponding author.

## Ethics Statement

The animal study was reviewed and approved by The University of Texas at Dallas Institutional Animal Care and Use Committee.

## Author Contributions

RM: writing – original draft and formal analysis. SA and TD: project administration. RM, SA, TD, VE, AS, and KA: investigation. RM, MK, and SH: conceptualization, writing – review and editing. MK, RR, and SH: supervision, funding acquisition, and review. All authors contributed to the manuscript revision and approved the submitted version.

## Conflict of Interest

MK has a financial interest in MicroTransponder, Inc., which is developing VNS for stroke. RR has a financial interest in Xnerve Medical, Inc., which is developing several VNS based therapies. The remaining authors declare that the research was conducted in the absence of any commercial or financial relationships that could be construed as a potential conflict of interest.

## Publisher’s Note

All claims expressed in this article are solely those of the authors and do not necessarily represent those of their affiliated organizations, or those of the publisher, the editors and the reviewers. Any product that may be evaluated in this article, or claim that may be made by its manufacturer, is not guaranteed or endorsed by the publisher.
