# Supplementary material for: Common Cholinergic, Noradrenergic, and Serotonergic Drugs Do Not Block VNS-Mediated Plasticity
Source: Front Neurosci. 2022 Feb 23;16:849291. doi: 10.3389/fnins.2022.849291 (PMC8904722; doi:10.3389/fnins.2022.849291)

# Common Cholinergic, Noradrenergic, and Serotonergic Drugs Do Not Block VNS-Mediated Plasticity

**Figure S1.**

Raw maps from ICMS. Axes denote stereotaxic coordinates relative to bregma with rostro-caudal directions from +5 to -4 mm and mediolateral directions from 0 to +6 mm. Movement thresholds are displayed in μA.

**Figure S2**

(A) Average movement thresholds from ICMS for all responses. (B) Average movement thresholds from ICMS for jaw responses only. No change in stimulation thresholds were found between groups. Bars represent mean ± SEM.

# Figure S1

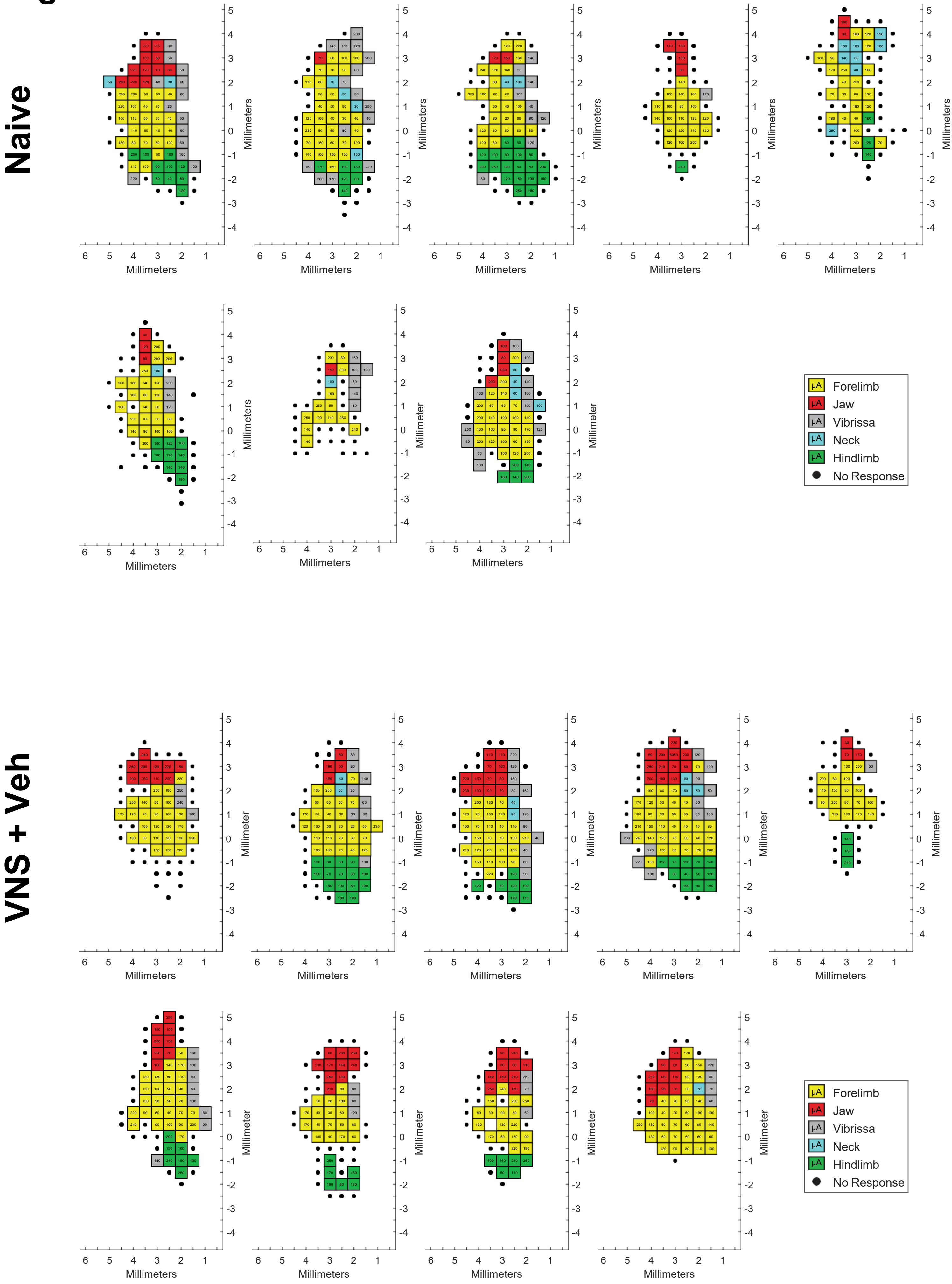

Naive
VNS + Veh
Millimeters
Millimeter
μA Forelimb
μA Jaw
μA Vibrissa
μA Neck
μA Hindlimb
No Response

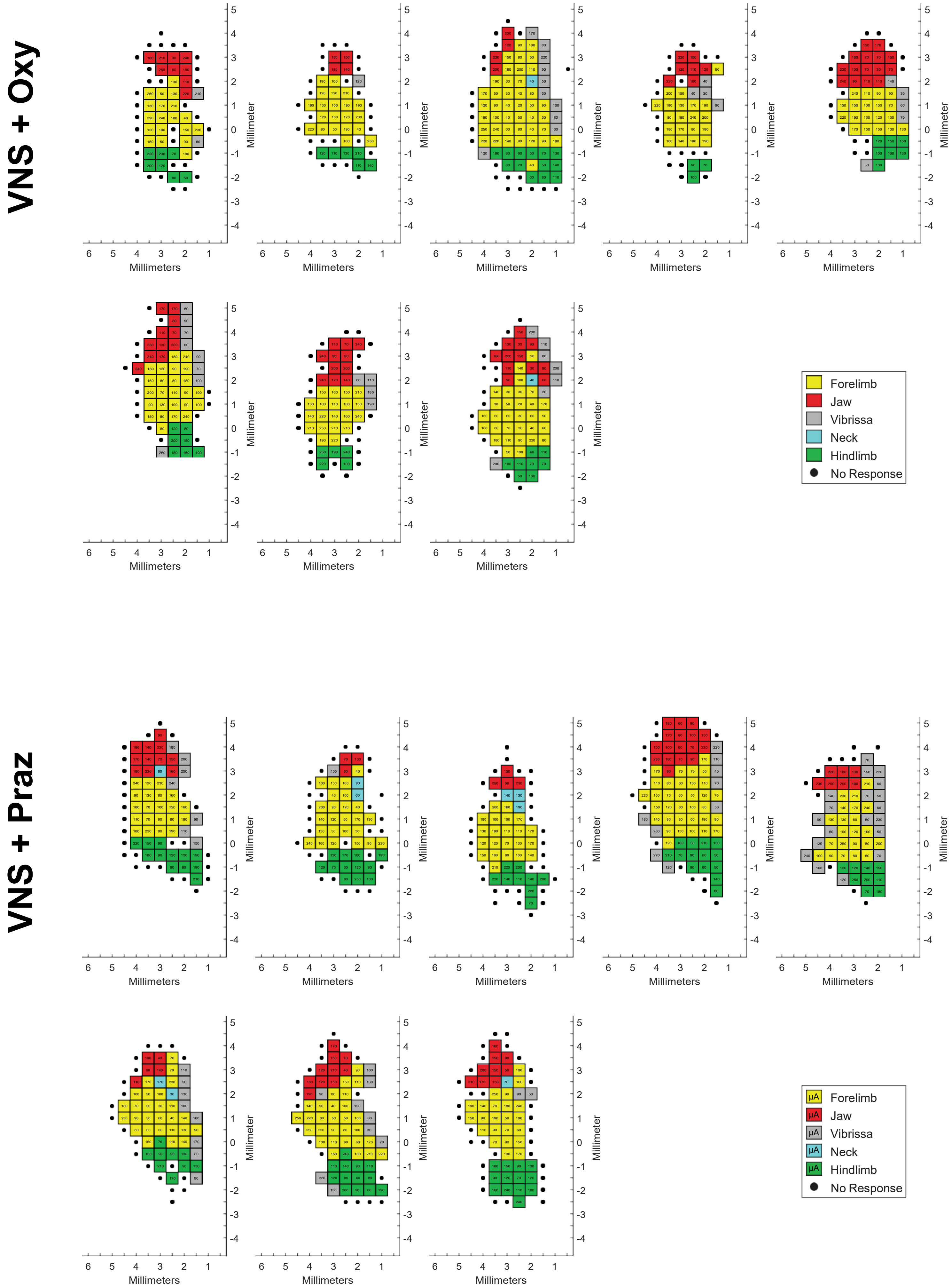
VNS + Oxy
VNS + Praz
Millimeters
Millimeter
Forelimb
Jaw
Vibrissa
Neck
Hindlimb
No Response
µA

**VNS + Dulox**

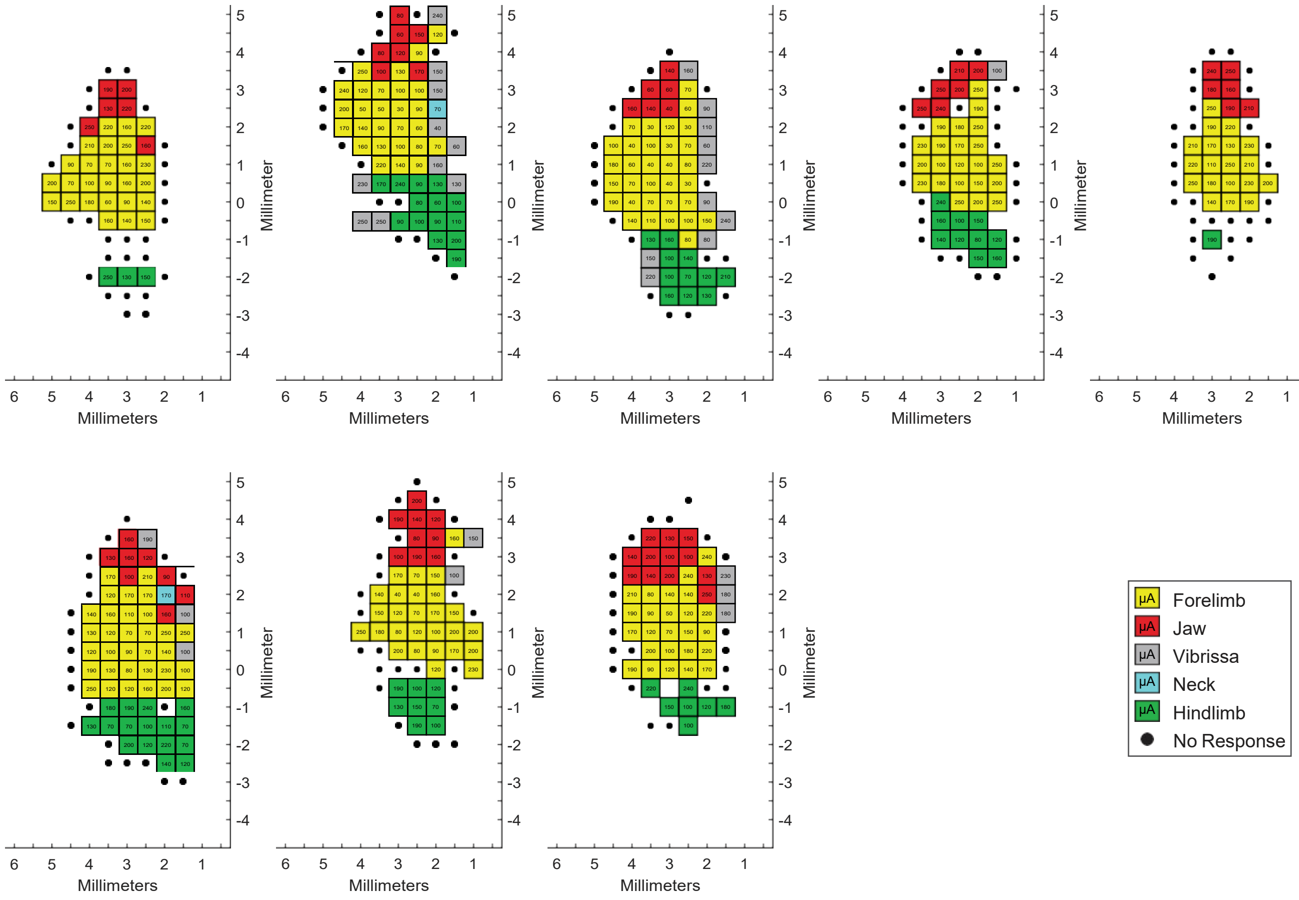


# Figure S2

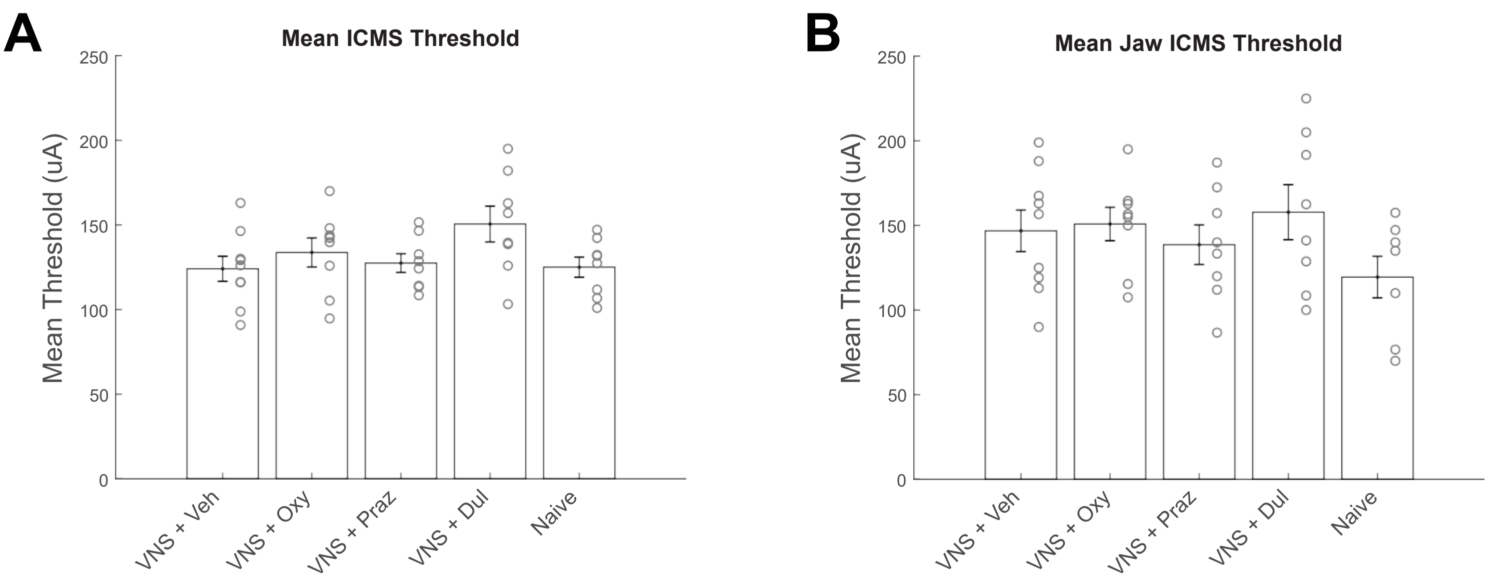

Supplement: Supplementary file 1 [file Data_Sheet_1.pdf]
